# Informing the scale-up of Kenya’s nursing workforce: a mixed methods study of factors affecting pre-service training capacity and production

**DOI:** 10.1186/1478-4491-12-47

**Published:** 2014-08-20

**Authors:** Ashley A Appiagyei, Rose N Kiriinya, Jessica M Gross, David N Wambua, Elizabeth O Oywer, Andrew K Kamenju, Melinda K Higgins, Patricia L Riley, Martha F Rogers

**Affiliations:** 1 Emory University Rwanda Zambia HIV Research Group, Lusaka, Zambia; 2 Emory University Kenya Health Workforce Project, Nairobi, Kenya; 3 Nursing Council of Kenya, Nairobi, Kenya; 4 Emory University Nell Hodgson Woodruff School of Nursing, Atlanta, GA, USA; 5 Division of Global HIV/AIDS, Centers for Disease Control and Prevention, Center for Global Health, Atlanta, GA, USA

**Keywords:** Nursing workforce, Kenya, Training, Scale-up

## Abstract

**Background:**

Given the global nursing shortage and investments to scale-up the workforce, this study evaluated trends in annual student nurse enrolment, pre-service attrition between enrolment and registration, and factors that influence nurse production in Kenya.

**Methods:**

This study used a mixed methods approach with data from the Regulatory Human Resources Information System (tracks initial student enrolment through registration) and the Kenya Health Workforce Information System (tracks deployment and demographic information on licensed nurses) for the quantitative analyses and qualitative data from key informant interviews with nurse training institution educators and/or administrators. Trends in annual student nurse enrolment from 1999 to 2010 were analyzed using regulatory and demographic data. To assess pre-service attrition between training enrolment and registration with the nursing council, data for a cohort that enrolled in training from 1999 to 2004 and completed training by 2010 was analyzed. Multivariate logistic regression was used to test for factors that significantly affected attrition. To assess the capacity of nurse training institutions for scale-up, qualitative data was obtained through key informant interviews.

**Results:**

From 1999 to 2010, 23,350 students enrolled in nurse training in Kenya. While annual new student enrolment doubled between 1999 (1,493) and 2010 (3,030), training institutions reported challenges in their capacity to accommodate the increased numbers. Key factors identified by the nursing faculty included congestion at clinical placement sites, limited clinical mentorship by qualified nurses, challenges with faculty recruitment and retention, and inadequate student housing, transportation and classroom space. Pre-service attrition among the cohort that enrolled between 1999 and 2004 and completed training by 2010 was found to be low (6%).

**Conclusion:**

To scale-up the nursing workforce in Kenya, concurrent investments in expanding the number of student nurse clinical placement sites, utilizing alternate forms of skills training, hiring more faculty and clinical instructors, and expanding the dormitory and classroom space to accommodate new students are needed to ensure that increases in student enrolment are not at the cost of quality nursing education. Student attrition does not appear to be a concern in Kenya compared to other African countries (10 to 40%).

## Background

Human resources for health (HRH) are a fundamental component of health system strengthening. Sub-Saharan Africa has a shortage of skilled health care workers [1,2], with 25% of the world’s disease burden and only 3% of the world’s trained health workforce [1]. The 2006 World Health Report [1] and the Joint Learning Initiative [3] described the positive correlation between the health workforce supply and improved health services delivery. Globally, maternal and child survival are linked to the density of skilled health workers.

The 2006 World Health Assembly highlighted the need to rapidly scale-up the health workforce in sub-Saharan Africa, calling global heath partners to support training institutions to improve their capacity to provide quality health professional education in low-income countries [4]. The 2013 World Health Assembly passed a resolution supporting transformative health worker education, noting the need for comprehensive situation assessments of member states’ current capacity related to health worker education to inform needed improvements to health workforce education and training systems [5].

The First Global Forum on HRH in 2008 produced *The Kampala Declaration and Agenda for Global Action*[6] - which recognized the HRH crisis and the devastating impact of HIV/AIDS and other diseases on health systems. The declaration calls countries to develop health workforce information systems, to ‘improve research and to develop capacity for data management in order to institutionalize evidence-based decision-making and enhanced shared learning’ [6], as well as for governments to determine the ‘appropriate skill mix and institute coordinated policies…for an immediate, massive scale-up of community and mid-level health workers’ [6].

The *Agenda for Global Action* identified several strategies to strengthen the health workforce, including scaling-up health worker education and training monitored by health workforce information systems [6]. This commitment to scaling-up health worker education and training was renewed at the Second Global Forum on HRH in 2011 [7] and the World Health Organization launched related guidelines at the Third Global Forum on HRH in 2013 [8]. While previous efforts to scale-up the health workforce have targeted select areas of the HRH supply and deployment pipeline, depending on stakeholder activities and donor interests, efforts to address all stages of the production pipeline as a chain of interrelated issues are lacking [9,10].

In 2008, the United States President’s Emergency Plan for AIDS Relief (PEPFAR) committed to support the education and training of 140,000 new health care workers in recipient countries by 2014, with an emphasis on doctors, nurses, and midwives [11]. Other initiatives and organizations, including the Global Alliance for Vaccines and Immunization (GAVI), the Global Fund to Fight AIDS, Tuberculosis, and Malaria (GFATM), the Clinton Foundation, and the Japan International Cooperation Agency (JICA) [1,12], have also made efforts to facilitate the scale-up of health worker education and training.

Kenya’s workforce density is below the threshold needed to meet health related Millennium Development Goals [1]. In Kenya, nurses provide the bulk of health care services. In Northeastern Province, where health outcomes related to maternal health fall behind the rest of the country [13], the public sector nurse to population ratio is 24 to 100,000 compared to Nairobi Province where the ratio is 95 to 100,000 with relatively better health outcomes [14]. To address Kenya’s nursing shortage, the Government of Kenya (GOK) prioritized increasing the health workforce capacity nationally [15].

The 2010 *Lancet* report, *Health Professionals for a New Century: Transforming Education,* notes that investments in pre-service nursing education should be considered within the framework of the health system, such that regulation, education, training, research and service delivery work synergistically, and not in isolation [16-18]. In collaboration with country-led health workforce development plans, capacity building for nurse training institutions is required [7,12] to ensure the pipeline from professional enrolment, or indexing, of new students into training through to recruitment into the health system is maximized to support the scale-up [16].

Although Kenya’s HRH strategic plan does not set specific workforce production goals, the GOK is committed to enhancing health workforce training [19]. As of 2012, the Nursing Council of Kenya (NCK), which regulates the education for nurses in Kenya, reported that Kenya had 82 accredited nurse training institutions. Ten of these 82 institutions opened between 2010 and 2012. Of the 82 institutions, 19 are in Rift Valley, 15 in Central, 15 in Nairobi, 10 in Eastern, 9 in Nyanza, 6 in Western, 5 in Coast and 3 in North Eastern Province. Together these institutions offered 5 enrolled programmes, 60 registered programmes and 19 BScN programmes across Kenya (E Oywer, Registrar, NCK, personal communication, 24 October 2012). School fees vary with an annual cost of $700 to $900 at GOK institutions and $1,500 for faith-based and private institutions. Programme length varies by type with basic enrolled training lasting two and a half years, registered three to three and a half years and BScN four years plus a one-year internship. All graduates are required to pass an NCK licensing exam, which they can retake if needed, prior to compulsory registration with the council to practice as a nurse in Kenya (E Oywer, Registrar, NCK, personal communication, 26 November 2013).

Strategies aimed to increase the nursing workforce must ensure related investments in the capacity of pre-service training institutions, including faculty development and educational infrastructure [12]. Training institutions in low-income countries are struggling financially and many donors and development agencies lack coherent and integrated investment strategies to strengthen the workforce, resulting in an overemphasis on workshops and training sessions that have an indeterminate effect [20]. Instead of strengthening training institutions and supporting faculty development, training capacity in many low-income countries remains inadequate [21]. Efforts that fail to expand faculty capacity and support faculty advancement can negatively impact student performance and teaching staff retention [22]. Significant investment will be required to strengthen educational infrastructure, faculty and staff development, curriculum review and clinical instruction [12,17,21,23].

Student attrition during pre-service education should also be evaluated to inform scale-up planning. In four sub-Saharan African countries, pre-service training student attrition ranged from 10 to 40% [24]. In order to maximize returns on investments in pre-service education and training, it is essential that governments track individuals from enrolment to licensure and employment to enhance the output of newly qualified nurses to meet the health care demands. While studies have examined the attrition of nurses in Kenya’s public sector [15,25], no published research has investigated the attrition of student nurses prior to joining the workforce. Understanding the progression from pre-service enrolment to registration with the council is important to inform the workforce scale-up.

In order for the GOK and donor agencies to strategically invest in expanding pre-service nursing education to scale-up Kenya’s health workforce, accurate training capacity and student attrition data is required to support evidenced-based strategies to increase the number of licensed and registered nurses. To that end, this study evaluated (1) trends in annual student nurse enrolment, (2) the level of pre-service attrition between enrolment and registration with the NCK, and (3) factors that influence training institutions’ capacity to produce nurses as identified by nursing faculty.

## Methods

**Figure 1:**
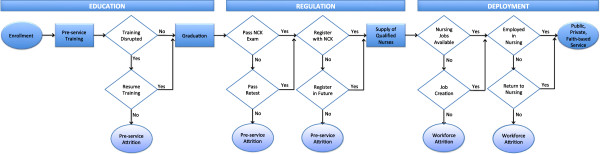
Framework of supply and deployment pipeline for nursing in Kenya.

This study used a mixed methods approach to investigate the three study aims to inform efforts for scaling-up Kenya’s nursing workforce. Figure 1 illustrates the stages in the nurse education, regulation and deployment in Kenya and how losses at each stage result in fewer trained and deployed nurses. This study analyzed data related to nursing education and regulation to better understand factors that influence nurse production. Analysis of deployment data was beyond the scope of this study.

The quantitative component of this study analyzed de-identified nursing data from the Regulatory Human Resources Information System (rHRIS), a family of databases designed for the regulatory agencies of each health professional cadre in Kenya. The nursing component of the rHRIS houses regulatory information (for example, enrolment, training and licensure) and is complimented by the Kenya Health Workforce Information System (KHWIS), another database capturing mostly public health sector deployment information on health workers (for example, demographic information, current location and health facility type). The rHRIS is managed by the nursing regulatory agency - the NCK, while the KHWIS is managed by the Department of Nursing (DON), Ministry of Health (MOH). Other studies have been published on the development [26] and impact [27] of the KHWIS.

De-identified data from the rHRIS and KHWIS for this study was formatted in Microsoft Access, cleaned and analyzed using statistical software (STATA©, StataCorp., College Station, TX, USA) by project analysts familiar with data collection and coding for these systems. Permission to analyze this data was granted by the NCK and MOH. In addition, Kenyan educators and administrators granted permission to conduct in-person interviews. The study protocol was also reviewed and approved by the Emory University Institutional Review Board.

To assess trends in the annual enrolment of nursing students, data from the rHRIS and KHWIS was used to determine the annual number of enrolled students from 1999 to 2010. Student enrolment was analyzed by number of students per year, type of programme, training institution, sponsoring agent (for example, public, private), and demographic make up of the students, including gender and age. Enrolment is defined as the first stage in nurse production.To assess pre-service attrition between training enrolment and registration with the NCK, data for a cohort that enrolled in training from 1999 to 2004 was analyzed. 2004 was used as the cut-off to allow time for students to complete their course of study, which most students would have done by 2010. This interval was sufficient to document and analyze student movement through enrolment, graduation, licensure examination, and registration in the nurse supply pipeline, a process that takes two and a half to five years depending on the programme of enrolment (Figure 1) - or longer if the nurse experiences temporary training disruptions. Nurses were tracked using a unique identifier. Attrition was defined as a student that enrolled in nurse training but failed to register with the council to practice. Registration is defined as the final stage in nurse production.

The study cohorts included all individuals, 17 years of age and older, who enrolled in pre-service training for the first time in Kenyan nurse training institutions - public, private and faith-based. Nurses trained outside Kenya were excluded from all analyses since this study is limited to Kenyan institutions.

The attrition cohort was followed to quantify the loss of potential additions to the nurse workforce as a result of non-registration with the NCK. To examine factors associated with attrition or non-registration, nurses who registered were compared with those who failed to register using Pearson chi squared tests and multivariate logistic regression to determine if age, gender, home province, year of enrolment, training programme (that is certificate programme for enrolled nursing, diploma programme for registered nursing, or a degree programme conferring the BScN), training institution (that is public, private, faith-based), or province location of the training institution affected the likelihood of registration with the NCK.

To assess the capacity of nurse training institutions, qualitative information was obtained through key informant interviews (KIIs) with nurse training institution educators and/or administrators. Nurse training institution educators and/or administrators were selected as key informants because they oversee and coordinate the training of nursing students and are familiar with factors that affect pre-service nursing education. Using interview guides (Additional file 1), key informants were asked about potential barriers to scaling-up nurse training in Kenya, including perceived reasons for training disruptions and student attrition, challenges with students’ clinical experience and mentorship, as well as challenges experienced by faculty and lecturers.

The study population for the KIIs included a convenience sample of nine administrators and/or faculty from nine different nurse training institutions in four provinces - Nairobi, Central, Eastern and Rift Valley. The sample size was determined by conducting interviews until data saturation of themes was reached [28]. The sample included participants from six public institutions, two faith-based, and one private.

Guides for the KIIs were developed by authors from Emory University and reviewed and edited by collaborators from the Emory University Kenya Health Workforce Project KHWP and NCK. Data was collected in 2010 and analyzed using the qualitative data analysis software MaxQDA© in 2011. All qualitative data was coded and a thematic analysis was conducted to identify both recurrent and unique statements and themes.

## Results

### Trends in new student nurse enrolment

There were 23,350 students who enrolled in pre-service nursing training in Kenya between 1999 and 2010. New student enrolment doubled between 1999 (1,493 students) and 2010 (3,030 students). At initial enrolment, the mean age was 21 years with 27.1% aged 17 to 20 years, 65.3% aged 21 to 25 years, 6.2% aged 26 to 30 years and 1.4% over the age of 30. Females comprised 73.1% of new students and 26.9% were males.

Annual student nurse enrolment began to climb steadily in 2005 (1,652 students) with new student enrolment increasing by approximately 255 students annually over the previous year - between 2005 and 2010 - until it reached 3,030 new students in 2010. Most students training in Kenya were Kenyan (99.9%) with nearly a quarter being from Rift Valley province (24.8%). Additionally, the majority of new students were from Eastern (18.5%), Nyanza (16.1%), Central (15.3%) and Nairobi (14.0%) provinces with the fewest being from North Eastern (1.3%), Coast (4.4%) and Western (5.6%) provinces.

These students trained in 72 nurse training institutions across Kenya with the majority of students trained in Rift Valley (20.7%), Eastern (19.7%), Central (17.7%), Nyanza (14.7%) and Nairobi (12.0%) provinces. North Eastern, Coast and Western provinces trained new student nurses, but to a lesser extent, representing only 2.4%, 6.0% and 6.8% of new student enrolment respectively. Most nurses (73.6%) trained in diploma-conferring registered nursing programmes with 18.1% in certificate-conferring enrolled nursing programmes and 8.3% in degree-conferring BScN programmes. Due to the phasing out of enrolled nursing programmes to raise the standard of entry level nursing, new student enrolment in enrolled nursing programmes declined from 42.2% of new students in 1999 to 6.2% in 2010, while enrolment in BScN programmes climbed from 4.5% in 1999 to 10.0% in 2010.

Public institutions trained 62.9% of new students, followed by faith-based (32.8%) and private (4.3%) institutions. Government sponsored Kenya Medical Training Colleges (KMTCs) enrolled 54.1% of new nursing students at their 23 nurse training institutions, followed by the Consolata Nyeri School of Nursing (651), Consolata Nkubu School of Nursing (598), Nairobi Hospital School of Nursing (595), Baraton University (555), University of Nairobi (542) and Presbyterian Church of East Africa Tumutumu School of Nursing (526). From 1999 to 2010, GOK and private institutions increased their new student enrolment by 2.4 times and faith-based institutions increased theirs by 1.5 times.

### Factors that influence nurse production

#### I. Pre-service attrition between enrolment and registration

There were 8,959 students who enrolled in nurse training between 1999 and 2004. Of these, 8,436 (94%) completed their training, passed the NCK examination, and registered to practice nursing in Kenya between 2001 and 2010. Five hundred and twenty-three (6%) failed to register with the NCK. Pre-service training attrition doubled between students who enrolled in 1999 (4.3%) and 2004 (8.2%).

There were several differences between students who registered and those who did not (Table 1). Non-registering students were more likely to be older at the time they began pre-service training - 17.4% of students older than 30 years of age at enrolment failed to register compared to 5.5% for those aged 21 to 25 years. Students over 30 years of age at enrolment were 3.4 times less likely to register compared to students 21 to 25 years of age.

**Table 1:** **Registration versus non-registration among Kenyan nursing students**^**ab**^

	**Total (%)**	**Registered (%)**	**Non-registered (%)**	**Odds ratio**	***P*-value**	**95% CI**
Age Group						
17 to 20	1,993 (23.6)	1,880 (94.3)	113 (5.7)	0.953	0.678	0.758 to 1.197
21 to 25	5,757 (68.5)	5,443 (94.5)	314 (5.5)	Ref		
26 to 30	575 (6.8)	540 (93.9)	35 (6.1)	0.934	0.723	0.639 to 1.364
*> 30*	*92 (1.1)*	*76 (82.6)*	*16 (17.4)*	*0.298*	*0.000*	*0.160 to 0.555*
Gender						
Female	6,725 (75.5)	6,345 (94.3)	380 (5.7)	Ref		
Male	2,180 (24.5)	2,048 (93.9)	132 (6.1)	0.929	0.481	0.758 to 1.140
Home Province						
Coast	319 (3.6)	310 (97.2)	9 (2.8)	Ref		
Central	1,429 (16.2)	1,358 (95.0)	71 (5.0)	0.509	0.090	0.233 to 1.112
Nairobi	1,088 (12.4)	1,045 (96.0)	43 (4.0)	0.731	0.445	0.326 to 1.636
Eastern	1,528 (17.4)	1,460 (95.5)	68 (4.5)	0.559	0.147	0.255 to 1.226
North Eastern	160 (1.8)	154 (96.2)	6 (3.8)	0.486	0.208	0.158 to 1.496
*Nyanza*	*1,202 (13.7)*	*1,132 (94.2)*	*70 (5.8)*	*0.412*	*0.026*	*0.187 to 0.899*
*Rift Valley*	*2,340 (26.6)*	*2,190 (93.6)*	*150 (6.4)*	*0.409*	*0.021*	*0.191 to 0.876*
*Western*	*734 (8.3)*	*674 (91.8)*	*60 (8.2)*	*0.328*	*0.006*	*0.149 to 0.724*
Training Province						
North Eastern	237 (2.7)	229 (96.6)	8 (3.4)	ref		
Coast	413 (4.6)	394 (95.4)	19 (4.6)	0.541	0.209	0.207 to 1.412
Central	1,633 (18.2)	1,546 (94.7)	87 (5.3)	0.685	0.387	0.289 to 1.616
Nairobi	1,141 (12.7)	1,063 (93.2)	78 (6.8)	0.556	0.187	0.233 to 1.329
Eastern	1,591 (17.8)	1,536 (96.5)	55 (3.5)	0.939	0.888	0.391 to 2.256
Nyanza	1,222 (13.7)	1,158 (94.8)	64 (5.2)	0.874	0.765	0.362 to 2.112
Rift Valley	2,032 (22.7)	1,868 (91.9)	164 (8.1)	0.539	0.151	0.232 to 1.253
Western	687 (7.6)	639 (93.0)	48 (7.0)	0.541	0.179	0.220 to 1.327
Sponsoring Agent						
Public (GOK)	5,274 (58.9)	5,004 (94.9)	270 (5.1)	ref		
Private	401 (4.5)	379 (94.5)	22 (5.5)	0.799	0.415	0.465 to 1.371
*Faith-based*	*3,281 (36.6)*	*3,050 (93.0)*	*231 (7.0)*	*0.707*	*0.002*	*0.567 to 0.881*
Programme Type						
Enrolled	3,190 (35.6)	3,002 (94.1)	188 (5.9)	ref		
Registered	5,189 (57.9)	4,923 (94.9)	266 (5.1)	1.196	0.129	0.949 to 1.508
*BScN*	*580 (6.5)*	*511 (88.1)*	*69 (11.9)*	*0.621*	*0.008*	*0.436 to 0.885*
Enrolment Year						
1999	1,495 (16.7)	1,430 (95.7)	65 (4.3)	ref		
2000	1,466 (16.4)	1,398 (95.4)	68 (4.6)	1.012	0.950	0.701 to 1.460
2001	1,323 (14.8)	1,249 (94.4)	74 (5.6)	0.904	0.582	0.631 to 1.296
*2002*	*1,615 (18.0)*	*1,496 (92.6)*	*119 (7.4)*	*0.598*	*0.002*	*0.430 to 0.830*
2003	1,541 (17.2)	1,468 (95.3)	73 (4.7)	0.939	0.735	0.653 to 1.351
*2004*	*1,519 (16.9)*	*1,395 (91.8)*	*124 (8.2)*	*0.530*	*0.000*	*0.380 to 0.740*

^a^Note that due to missing data not all denominators are equal. Significant findings are noted in italics.

^b^Multivariate logistic regression includes all variables, except gender, which was not significant in the univariate analysis.

Non-registration varied according to the students’ home province. For example, non-registration was lowest among students from Coast (2.8%) and highest among students from Western (8.2%). Nursing students from Western, Rift Valley and Nyanza Provinces were 3.1, 2.5, and 2.4 times less likely to register, respectively, than students from Coast Province.

There was also a significant relationship between the institution sponsoring agent and non-registration. Among students trained in faith-based institutions, 7.0% did not register, compared to 5.1% of students trained in public institutions and 5.5% trained in private institutions. Students trained in faith-based institutions were 1.4 times less likely to register than students trained in public institutions.

Of the three training levels - enrolled, registered and BScN - BScNs had the highest proportion of non-registration at 11.9%, compared to 5.1% of diploma-conferred registered nurses and 5.9% of certificate-conferred enrolled nurses. Students training in BScN programmes were 1.6 times less likely to register than students in enrolled nursing programmes. Looking at trends over time, non-registration was the highest among individuals who began pre-service training in 2002 (7.4%) and 2004 (8.2%).

KII respondents cited several factors associated with training disruptions and student attrition, including pregnancy, insufficient money for school fees, and academic challenges. While pregnancy was the primary reason for training disruption, respondents reported that pregnant students did not drop out completely, but generally rejoined their current programme or continued with a subsequent class. Failure to pay fees was noted as a reason for training disruptions, but not for complete attrition. Self-sponsored students were identified as having the most difficulties in paying for school fees. All respondents cited academic difficulty or failure which typically resulted in students having to repeat a year before completing the programme. Disinterest among the students was also cited as a reason for academic failure, discontinuation and training disruptions. Training disruptions and student attrition was thought to be more common in the first year of training than subsequent years.

Overall, all respondents stated that student attrition was not more than 1 to 2% per year - consistent with the finding from the quantitative analysis of the KHWIS that 94% of students eventually registered. When asked about why a successful graduate might not register with the NCK, respondents noted that a lack of employment opportunities in nursing might contribute to non-registration of qualified graduates; however, all respondents stated that it was uncommon for graduates to move into non-health professions after completing the nursing programme.

#### II. Challenges with students’ clinical placements

While most institutions whose faculty was interviewed had a health facility annexed to their school as the primary site for students’ clinical experience, institutions also had to send students to other facilities, such as district hospitals and rural health facilities, to gain specific experience in specialties not offered at their facility, such as pediatrics, maternity and intensive care. These facilities were often used as clinical sites by nursing students from various institutions - public, private, and faith-based - as well as medical and clinical officer students, resulting in clinical site congestion.

Respondents stated that resources available in some teaching health facilities were often inadequate - inhibiting students from practicing clinical skills taught in the classroom. While some respondents noted that students were taught to use whatever resources were available to them, others stated that one of the advantages of the mentored clinical practice is that the students were exposed to the realities of limited resources in health facilities before entering the profession.

Due to the nursing shortage, respondents noted nurses were overworked and lack the necessary time to adequately supervise students. One private institution developed a mentorship programme for staff nurses responsible for mentoring students. Another respondent expressed the need to equip mentors with the necessary tools - such as up-to-date training materials - to teach students about emerging and re-emerging diseases. While some institutions, particularly the private and faith-based institutions, had clinical instructors to supervise and mentor students during their clinical experience, others lacked the necessary resources for clinical instructors.

When students have to travel to facilities outside of their institution for clinical experience, they confronted logistical challenges with transportation and accommodation problems. While some schools provided for transportation and/or accommodation at the clinical training site, other schools required students to pay for these expenses.

#### III. Tutor capacity

None of the institutions interviewed had the optimum faculty-to-student ratio of 1:10, recommended by the NCK. The ratios ranged from 1:14 in the private and faith-based institutions to as high as 1:40 in one public institution. In all public institutions interviewed, this resulted in a burdensome faculty workload and insufficient time for student clinical mentorship.

While there were many opportunities for continuing professional development (CPD), responses on institutions’ willingness to sponsor faculty to attend CPD were mixed. While some institutions adequately supported staff participation in CPD, faculty did not have time to attend the workshops, conferences or seminars. Other institutions did not have the financial resources to sponsor faculty participation in CPD. One respondent stated that his institution could only afford to send one or two out of twenty staff members. For long courses such as Masters’ degrees or PhD programmes, the majority of respondents said faculty was granted educational leave, but not financial support.

Some respondents stated that recruitment of staff was a problem for their institution. For the KMTCs, recruitment was carried out centrally, and faculty was often transferred between institutions depending on the need. One respondent noted faculty at their institution, which had a faculty-student ratio of 1:40, was transferred to other institutions that had even worse faculty-to-student ratios.

One respondent at a non-governmental institution highlighted a high turnover of faculty due to interest in moving into government positions, which were often better paying, contractual jobs. Salary and benefits, as well as location of the training institutions, were cited as factors contributing to the competitiveness in recruiting well-qualified faculty. Difficulty in retaining qualified faculty was also attributed to the age and level of training of faculty, as more experienced and educated faculty sought employment with better salaries and benefits.

#### IV. Limited Infrastructure

Many of the public institutions, particularly the KMTCs, were primarily established to train only one or two health professions. However, over time these institutions have expanded to accommodate additional professional training programmes without the requisite increase in resources. This situation, which has resulted in limited classroom space and student accommodation, represents additional training challenges. One institution decided to skip enrolment one year due to limited accommodation, resulting in a high number of student transfers.

## Discussion

**Figure 2:**
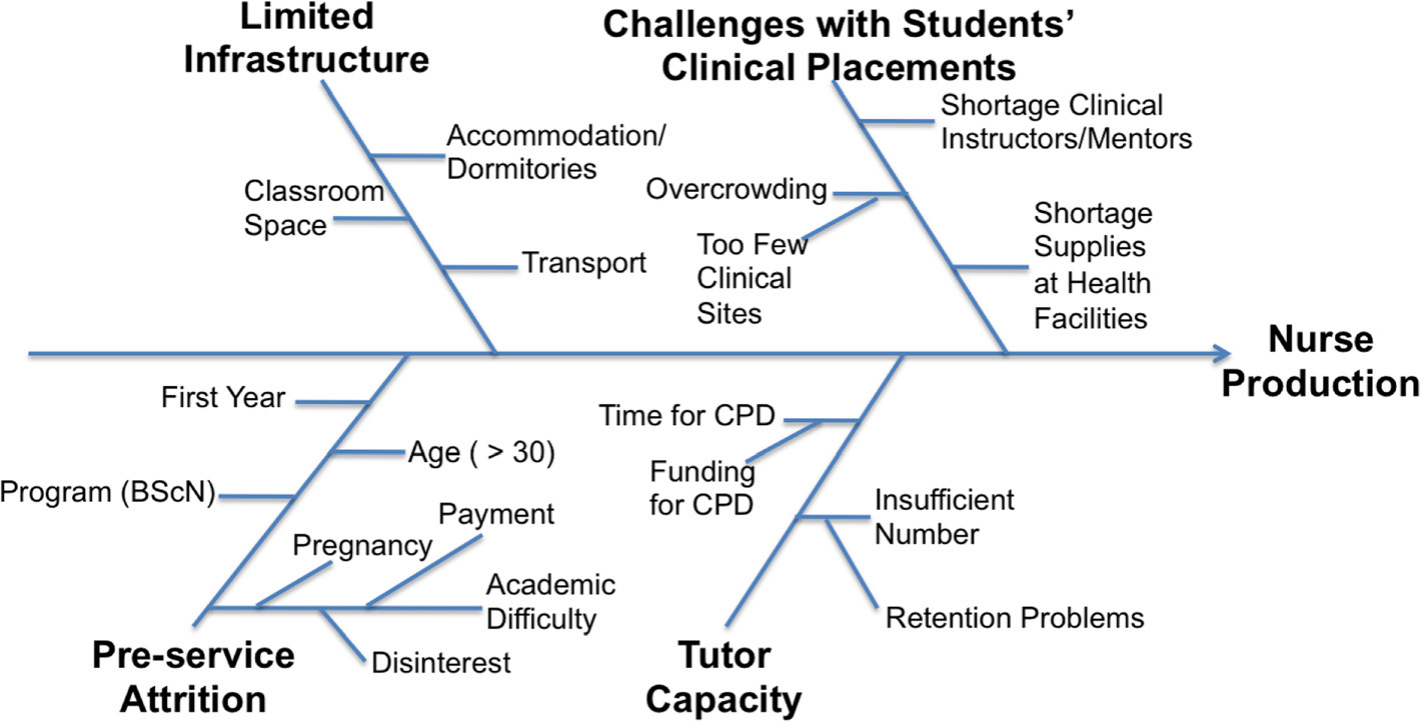
Fishbone diagram of identified factors influencing nurse production in Kenya.

As PEPFAR seeks to support Kenya’s scale-up of nurse training and education, this study identified specific factors that that influence nurse production - including challenges with students’ clinical placements, a shortage of nursing faculty, limited physical infrastructure and, to a more limited extent, pre-service attrition (Figure 2). While this study identified specific factors related to pre-service training attrition, such as non-registration with the council, other challenges were found to be much larger barriers to the scale-up of nurse training in Kenya. In order to maximize the return on public-private investment in nurse training in Kenya, interventions should explore the extent to which these factors limit the quantity and quality of nurse production addressing identified barriers and bottlenecks.

While Kenya has been able to increase annual new student enrolment in nursing training from 1,493 in 1999 to 3,030 in 2010, the KIIs have indicated that nurse training institutions were strained to accommodate the increase in numbers. Concurrent investments in expanding the number of student nurse clinical placement sites, utilizing alternate forms of skills training, hiring more faculty and clinical instructors, and expanding the dormitory and classroom space to accommodate new students are needed to ensure that increases in student enrolment are not at the cost of quality nursing education.

The KIIs identified problematic issues with student nurses’ clinical experiences, including inadequate student mentorship and clinical site congestion. Some studies from high-income countries have suggested that mentorship by qualified nurses during their clinical experience strongly influences student perceptions of the clinical learning environment [10,29,30]. In order to scale-up nurse training in Kenya, investing in clinical instructors who can fill in the gaps left by inadequate mentorship at clinical sites should be considered.

The NCK sets standards for clinical placements and approves clinical practice sites. To address the issue of congestion during students’ clinical experience, training institutions should work with the NCK to approve additional sites for clinical placement. Obtaining approval for new sites requires that specific standards be met related to student transport and accommodation, as well as patient load, to ensure a positive clinical experience for nursing students. Training institutions should also consider the increased use of skills labs that simulate the clinical practice environment with patient models.

High student to faculty ratios have resulted in overworked faculty that are unable to provide optimal student support. To address this issue, one institution established two- to three-year contracts for all newly employed faculty in order to enhance retention, which serves as a good model for other institutions. Also, schemes such as the faculty retention programme launched in Malawi [31], aimed at attracting and retaining nurse faculty by providing adequate incentives and opportunities for career advancement, should be considered. Interventions to attract and retain nurse faculty should consider increasing sponsorships for faculty to attend CPD training.

Compared to other sub-Saharan African countries that experience pre-service attrition between 10 and 40% [24], this study found that pre-service student nurse attrition in Kenya was low (6%). Training institution faculty and administrators indicated that training disruptions and student attrition were most likely due to pregnancy, financial difficulties, academic difficulty, and a disinterest in nursing. Improved counselling - including student guidance regarding reproductive health - enhanced access to financial assistance and greater academic support could address these issues. Further research is needed to evaluate if pre-service attrition continues to increase with subsequent increases in new student enrolment.

In this study, age at enrolment and programme type were found to significantly affect registration with the NCK. Students over the age of 30 at enrolment were 3.4 times less likely to register than students of 17 to 20 years of age and KIIs indicated that mature students often had more competing responsibilities, including family and financial obligations. Studies from the UK [10] and the US [32] have suggested that mature students are more likely than younger students to experience temporary and permanent student attrition.

Students trained in BScN programmes were most likely to have failed to register with the NCK. BScN nurses receive the highest level of pre-service training, which requires a one-year internship beyond classroom training. In an effort to improve the number of BScN graduates that successfully complete their required one-year internship to register with the NCK, the GOK began taking 200 BScN interns per year in 2009, which were given a stipend (S Ateka, Human Resources Department, MOH, personal communication, 4 September 2013). Further research is needed to understand if their degree enables them to migrate or obtain employment in other roles that do not require registration with the council - such as administration and project management in public health organizations - compared to enrolled or registered nurses.

Finally, scaling-up nurse production will make little difference if newly registered nurses cannot find employment in Kenya. The KIIs cited lack of employment opportunities in the nursing field as a possible reason why successful graduates fail to register or seek employment elsewhere. Limitations in public sector hiring due to budgetary constraints have been cited as reasons for migration and unemployment among nurses in Kenya in the past [1,25,33]. In line with national HRH strategic plans, the public, private and development sectors must work together to create nursing jobs to which newly trained and registered nurses can be recruited and deployed (Figure 1).

### Study limitations

Although the NCK regulatory data system captures accurate data on new student enrolment (indexing) and registration, the collection of data at other points in the pipeline is limited (for example, graduation, exam performance). Due to limited funding, a convenience sample of institutions in close proximity to Nairobi was selected for KIIs and the number of interviews was determined by saturation in data themes. To minimize any bias, the authors included at least one institution from public, private, and faith-based sectors and respondents varied in regards to urban versus rural institutions. This study was not able to interview nursing students who experienced training disruptions or nurses who failed to register with the NCK. Further research is needed to understand the factors causing such large variation in tutor-to-student ratios between nurse training institutions, factors contributing to Kenya’s low level of pre-service attrition, and factors affecting Kenya’s ability to recruit, hire and deploy these new nurses.

## Conclusions

In order to successfully scale-up pre-service training and education for nurses in Kenya, this study identified several areas that must be taken into consideration. While new student nurse enrolment doubled between 1999 and 2010, this study found pre-service attrition between enrolment and registration to be low but noted training institutions were strained by the increased numbers in terms of having adequate faculty capacity, clinical sites and mentorship, and student housing and classroom space. Interventions to scale-up the nursing workforce must address potential bottlenecks in the scale-up of nursing training and education, allocating sufficient resources to facilitate students’ clinical experience, expanding the number of clinical sites, ensuring faculty capacity for student mentorship, increasing the number of clinical instructors, and addressing logistical challenges pertaining to transportation and accommodation for clinical rotations. Interventions should also improve the recruitment and retention of faculty, enhance their access to CPD and provide space for didactic instruction. These investments will support not only an increased quantity of new students, but also the quality of their pre-service education.

## Abbreviations

AIDS: Acquired Immunodeficiency Syndrome; ASPPH: Association of Schools and Programs of Public Health; BScN: Bachelor of Science in Nursing; CDC: Centers for Disease Control and Prevention; CI: Confidence Interval; CPD: Continuing Professional Development; DON: Department of Nursing; GA: Georgia; GAVI: Global Alliance for Vaccines and Immunizations; GFATM: Global Fund to Fight AIDS, Tuberculosis and Malaria; GOK: Government of Kenya; HIV: Human Immunodeficiency Virus; HRH: Human Resources for Health; JICA: Japan International Cooperation Agency; KHWIS: Kenya Health Workforce Information System; KHWP: Kenya Health Workforce Project; KII: Key Informant Interviews; KMTC: Kenya Medical Training Colleges; NCK: Nursing Council of Kenya; MOH: Ministry of Health; PEPFAR: President’s Emergency Plan for AIDS Relief; PhD: Doctor of Philosophy; rHRIS: Regulatory Human Resources Information System; UK: United Kingdom; USA: United States of America.

## Competing interests

The authors declare that they have no competing interests.

## Authors’ contributions

AAA, MFR and JMG developed the study design. AAA, DNW, and EOO facilitated the research and reviewed the manuscript. RNK and MKH conducted the data analysis, assisted by AKK. AAA conducted the research and drafted the manuscript. PLR, MFR and JMG participated in the oversight for the study and manuscript review and revision. All authors read and approved the final manuscript.

## Authors’ information

Ashley Appiagyei is a couples’ voluntary HIV counselling and testing programme manager with Emory University Rwanda Zambia HIV Research Group, Lusaka, Zambia. Rose Kiriinya is a data analyst, Jessica Gross is a research communications officer, and Andrew Kamenju is a software engineer with the Emory University Kenya Health Workforce Project, Nairobi, Kenya. David Wambua is the Data Manager and Elizabeth Oywer is the Registrar of the Nursing Council of Kenya, Nairobi, Kenya. Melinda Higgins is an associate professor for research and Martha Rogers is research professor at Emory University Nell Hodgson Woodruff School of Nursing, Atlanta, GA, USA. Patricia Riley is a senior nurse midwife with the Centers for Disease Control and Prevention, Center for Global Health, Division of Global HIV/AIDS, Atlanta, GA, USA.

## Supplementary Material

Additional file 1 Key Informant Interview Guides. Click here for file
