# Supplementary material for: Informing the scale-up of Kenya’s nursing workforce: a mixed methods study of factors affecting pre-service training capacity and production
Source: Hum Resour Health. 2014 Aug 20;12:47. doi: 10.1186/1478-4491-12-47 (PMC4142461; doi:10.1186/1478-4491-12-47)
Supplement: Additional file 1 — Key Informant Interview Guides. [file 1478-4491-12-47-S1.doc]

**Additional file 1: Interview Guides**

**LECTURERS’ & ADMINISTRATORS INTERVIEW GUIDE**

**What is the demographic make up of most of the students in your institution?**

*Domains: Age, gender, province of origin, previous education.*

**Probe 1:** What is the average age of the students who enter?

**Probe 2:** How does the age distribution differ by cadre?

**Probe 3:** What would you estimate is the ratio of males to females among the nursing students?

**Probe 4:** Do students at your school predominantly originate from certain provinces? Which ones?

**Probe 5:** Could you describe how students from certain provinces may have a different experience within the program versus others? Any patterns in what cadres they enroll in?

**Probe 6:** What is the previous educational status (level, type of institution, etc.) of the students in this institution? Any patterns in what cadres they enroll in?

**Can you please describe some of the main causes of training disruption in the nursing students in your institution.**

*Domains: Relationships between occurrence/causes of training disruption, and age, gender, province of origin, previous education, cadres, temporary versus permanent attrition*

**Probe 1:** What are some common causes of training disruption that you witness at your institution?

**Probe 2:** Is training disruption more common among older students versus younger students?

**Probe 3:** Is training disruption more common among male students versus female students?

**Probe 4:** Are certain types of disruption more associated with students from certain provinces?

**Probe 5:** Do you feel that the students’ previous education before entering the nursing program is associated at all with whether they experience training disruption?

**Probe 6:** Do you see training disruption in some cadres more than others?

**Probe 7:** Could you talk a bit about students who resume training after disruptions, if they are not expelled? How often does this happen?

**Probe 8:** For students who do resume training after disruptions, on average, how much time do they spend out of school? How does this differ between the different causes of disruption?

**Can you please tell me a bit about graduation from the nursing program, and subsequent application for, and performance in the NCK exam?**

*Domains: Time frame, how many students successfully graduate from the program, barriers to applying for the NCK exam, NCK exam performance.*

**Probe 1:** What would you estimate is the percentage of students who successfully complete the program in comparison to the number that enroll?

**Probe 2:** How does this differ by age group, gender, cadre, province of origin, previous education, whether or not they had training disruption?

**Probe 3:** What is the time frame between graduation from the nursing program, and application for/ administration of the NCK exam?

**Probe 4:** What proportion of students apply for the exam upon successfully completing the program?

**Probe 5:** Have there been any instances where you have known students who completed the program but did not apply for the NCK exam? Reasons?

**Probe 6:** Have there been any instances where you have known students who applied for the exam but did not sit for them? Reasons?

**Probe 7:** How common is it for students to fail the exam on the first try?

**Probe 8:** What proportion would you say retakes the exam after failing the first time?

**NCK PERSONNEL INTERVIEW GUIDE**

**Can you please describe the process students go through in order to apply for the NCK exam?**

*Domains: Time frame, ease of application, barriers to NCK exam application*

**Probe 1:** What is the time frame between graduation from the nursing program, and application for/ administration of the NCK exam?

**Probe 2:** What would you estimate is the number of exam applications you receive each year?

**Probe 3:** Are there ever any instances where students who apply to take the exam are not allowed to?

**Probe 4:** What proportion of students who apply to take the exam, actually end up sitting for it?

**Probe 5:** Why do you think some students who have applied for the exam do not sit for them?

**What patterns if any have you witnessed with performance in the NCK exam?**

*Domains: Relationships between NCK exam performance and age, gender, (province of origin and previous education – if they know), training facility name/type/location.*

**Probe 1:** What proportion of students who sit for the exams, pass them?

**Probe 2:** Are there any nursing schools that have significantly higher student pass rates than others?

**Probe 3:** Have you noticed any relationships between good performance on the exam and age or gender? (and province of origin and previous education – if they know).

**Probe 4:** How common is it for students to fail the exam on the first try?

**Probe 5:** What proportion would you say retakes the exam after failing the first time?

**Can you please describe the process students go through to register as a nurse with the NCK after they successfully pass the NCK exam?**

*Domains: Time frame, ease of registration, barriers to registration*

**Probe 1:** How long does it take after taking the exam for students to obtain their results?

**Probe 2:** What proportion of students who pass the exam subsequently register with the NCK?

**Probe 3:** What are some reasons that students who pass the exam might not register with the NCK?
